# TXNRD2 (rs35934224) CT genotype as possible protective marker for primary open-angle glaucoma in a Brazilian population

**DOI:** 10.5935/0004-2749.20220019

**Published:** 2025-08-21

**Authors:** Artur Lins Tenório, Rodrigo Pessoa Cavalcanti Lira, Rodrigo Feliciano do Carmo, Roberto Pedrosa Galvão Filho, Pedro Teixeira Falcão Neto, Rinalva Tenório Vaz, Raul Emidio de Lima, André Lins Tenório, Luydson Richardson Vasconcelos

**Affiliations:** 1 Programa de Pós-graduação em Cirurgia, Universidade Federal de Pernambuco, Recife, PE, Brazil; 2 Universidade Federal do Vale do São Francisco, Petrolina, PE, Brazil; 3 Instituto de Olhos do Recife, Recife, PE, Brazil; 4 Hospital de Olhos Santa Luzia, Recife, PE, Brazil; 5 Instituto Aggeu Magalhães, Campus da Universidade Federal de Pernambuco, Recife, PE, Brazil; 6 Faculdade de Ciências Médicas, Universidade de Pernambuco, Recife, PE, Brazil

**Keywords:** Polymorphism, Thioredoxin reductase, Glaucoma, open-angle, Polimorfismo, Tiorredoxina redutase, Glaucoma de ângulo aberto

## Abstract

**Purpose:**

To investigate the association of the single-nucleotide polymorphism rs35934224 in the *TXNRD2* gene with primary open-angle glaucoma in a Brazilian population.

**Methods:**

This was a cross-sectional study conducted to verify the association between the rs35934224 *TXNRD2* (thioredoxin reductase 2) and primary open-angle glaucoma in a population from the Northeast region of Brazil. A total of 184 individuals were enrolled, including 94 with primary open-angle glaucoma (45 men and 49 women) and 94 controls (40 men and 54 women) from the Recife Eye Institute.

**Results:**

The mean age was 68.85 years for the patients with glaucoma and 68.55 years for the controls. Genomic DNA was isolated using commercially available kits, and single-nucleotide polymorphism was detected with real-time polymerase chain reaction using TaqMan probes. The studied population was in Hardy-Weinberg equilibrium. The CT genotype was associated with protection against primary open-angle glaucoma (p=0.022).

**Conclusion:**

Our data suggest an association between *TXNRD2* gene polymorphism (rs35934224) with primary open-angle glaucoma in an admixed Brazilian population. This is the first study to investigate this single-nucleotide polymorphism in Latin American individuals with primary open-angle glaucoma.

## Abstract (pt)

**Objetivo:**

Investigar a associação do Polimorfismo de nucleotídeo único rs35934224 no gene *TXNRD2* com o glaucoma primário de ângulo aberto em uma população brasileira.

**Métodos:**

Tratou-se de um estudo transversal para verificar a associação entre o rs35934224 do *TXNRD2* (tioredoxina redutase 2) e o glaucoma primário de ângulo aberto em uma população da região nordeste do Brasil. Foram incluídos 184 indivíduos: 94 casos com glaucoma primário de ângulo aberto (45 homens e 49 mulheres) e 94 controles (40 homens e 54 mulheres) do Instituto de Olhos de Recife.

**Resultados:**

A idade média foi de 68,85 anos para os pacientes com glaucoma e 68,55 anos para os controles. O DNA genômico foi isolado usando kits comerciais disponíveis e a detecção do polimorfismo de nucleotídeo único foi realizada por PCR em tempo real usando sondas TaqMan. A população estudada estava em equilíbrio de Hardy-Weinberg. O genótipo CT foi associado à proteção ao glaucoma primário de ângulo aberto (p=0,022).

**Conclusão:**

Nossos dados sugerem uma associação entre o polimorfismo do gene *TXNRD2* (rs35934224) e o glaucoma primário de ângulo aberto em uma população brasileira. Este é o primeiro estudo a investigar esse polimorfismo de nucleotídeo único em indivíduos com glaucoma primário de ângulo aberto latino-americanos.

## INTRODUCTION

Primary open-angle glaucoma (POAG) is characterized by loss of retinal ganglion cells, optic nerve damage, and consequently, irreversible loss of the visual field^(1)^. An estimated 35-58 million people are affected by POAG worldwide, and this number is expected to increase up to 53 to 65.5 million by 2020 owing to the aging world population^(2-3)^.

Although many mechanisms have been proposed, the pathophysiology of POAG and the factors that contribute to the worsening of the disease still remains obscure^(4)^. Recent advances in genomic technologies and genome-wide association studies (GWAS) have greatly accelerated the discovery and understanding of genes and genomic regions associated with POAG and influence the quantitative endophenotype traits related to POAG pathogenesis^(5)^. However, most studies have been conducted in European and Asian populations. Data regarding the influence of these genes in other populations are lacking.

*TXNRD2* is on chromosome 22q11.21 and consists of 18 exons of relatively small size, with intron sizes ranging from 200 and 10,000 bp^(6,7)^. In addition, *TXNRD2* codes for thioredoxin reductase 2, a mitochondrial protein necessary for reducing the damage of reactive oxygen species (ROS) generated by oxidative phosphorylation and other mitochondrial functions^(8,9)^.

The importance of studying the single-nucleotide polymorphism (SNP) in the thioredoxin reductase 2 (*TXNRD2*) gene (rs35934224) is due the strong association found between this SNP and POAG in a recent GWAS^(6)^. In this study, Bailey et al. found a significant association of the T variant with POAG in European, Asian, and Oceanian populations, showing the protection of the T allele in relation to POAG (p=4.05 × 10^-11^)^(6)^. In addition to POAG, the genetic variation in *TXNRD2* was found to be associated with diabetic retinopathy^(10)^, prostate cancer^(11,12)^, gastric cancer^(13)^, and colon and rectal cancers^(14)^.

Whether this genetic protective factor is also found in more mixed-race Latin American populations is not yet known. Northeastern Brazil has a highly miscegenated population. Therefore, the aim of this study was to investigate the association of the SNP rs35934224 in the *TXNRD2* gene with POAG in this population.

## METHODS

### Study population

For the present study, individuals from Northeast Brazil who attended the Fundação Ação Visual/IOR in the city of Recife, Pernambuco, were recruited. The participants underwent a comprehensive ophthalmologic examination, which included a medical history review and slit-lamp biomicroscopy, gonioscopy, dilated fundoscopy examinations (90-dioptre lens; Volk Optical Inc., Mentor, OH, USA). Intraocular pressure (IOP) was assessed using a Goldmann applanation tonometer (Haag Streit AG, Bern, Switzerland). The standard automated perimetry was performed using the 24-2 Swedish Interactive Thresholding Algorithm (SITA) FAST of the Humphrey Field Analyzer II (Carl Zeiss Meditec, Inc., Dublin, CA, USA), and the peripapillary retinal nerve fiber layer (RNFL) thickness was measured on spectral-domain (SD) optical coherence tomography (OCT; Heidelberg Engineering, Heidelberg, Germany) to assess visual field defects or quantify glaucomatous damage in glaucoma patients, respectively.

A total of 94 individuals with POAG were included in the present study. POAG was diagnosed if glaucoma optic neuropathy (GON) and defects in the visual field or retinal nerve fiber layer thickness, regardless of the IOP in one or both eyes, were found, on the basis of the following three criteria: (1) morphological changes in the optic nerve head characteristic of GON, with a cutoff cup-to-disk ratio of 0.7 to distinguish GON from healthy eyes or an asymmetry ≥0.2 between eyes, and the presence of localized defects in the RNFL and/or neuroretinal rim area; (2) at least two OCT images showing progressive retinal nerve fiber layer thinning or visual field defects corresponding to the RNFL thinning in the standard automated perimetry, considering glaucomatous visual field defects with a pattern standard deviation outside the 95% normal limits or a glaucoma hemifield test result >99% (Humphrey Swedish Interactive 24-2 SITA-FAST protocol); and (3) open and normally appearing angle of the anterior chamber in gonioscopy.

POAG was diagnosed in the patients before enrollment in the study. Patients who were using eye drops other than hypotensive medications after any ocular surgery and had <6 months of treatment with any glucocorticosteroid or immunosuppressive drugs were excluded from the study.

The control group consisted of 94 individuals without glaucomatous optic nerve neuropathy, with a cup-to-disk ratio of up to 0.5 and an IOP <18 mmHg. None of the healthy controls had evidence of any ocular diseases or autoimmune/autoinflammatory and systemic disorders matched by geographical region. Furthermore, patients with a familial history of glaucoma or blindness were excluded.

Written informed consent was obtained from all the participants, and the ethics committee for research of the University of Pernambuco, under protocol No. 56467316.8.0000.5207, approved all the methods used. All the study methods were performed in accordance with the provisions of the Declaration of Helsinki and Good Clinical Practice guidelines.

### DNA extraction and genotyping

Genomic DNA was extracted from whole blood by using a QIAamp DNA Blood Kit (Qiagen Inc, Chatsworth, CA), following the manufacturer’s instructions. The extracted DNA was measured using Nanodrop spectrometry technology and stored at -20°C until further analysis.

A 7500 real-time PCR system (Applied Biosystems, Foster City, CA) was used to detect the SNP of the gene *TXNRD2* (rs35934224) by using the TaqMan probe (Assay: C___2539086_10). The assays were performed with a TaqMan genotyping master mix (Applied Biosystems, Foster City, CA) in 96-well plates.

### Statistical analyses

The statistical analysis of the data was performed using the SPSS version 22.0 statistical software package (SPSS, Inc., Chicago, IL, USA). Categorical variables were compared using the chi-square test, and the results are presented using odds ratios (ORs) with 95% confidence intervals (CIs). Sex and age variables were analyzed between the groups using the Student *t* test or Mann-Whitney *U* test for parametrically or non-parametrically distributed data as appropriate. The differences were considered statistically significant when the p value was <0.05.

## RESULTS

The study population was in Hardy-Weinberg equilibrium. Table 1 describes the mean age and sex distributions between the cases and controls. The frequencies of the males and females and the mean ages were similar between the cases and the controls (p>0.05).

**Table 1:** Mean age and sex distribution between the patients with POAG and the controls

		POAG group	Control group	p value
Total		94	94	
Age (years)	Mean ± SD	68,85 ± 15,3	68,55 ± 12,0	0,946
IOP (mmHg)	Mean ± SD	17,75 ± 5,70	13,36 ± 2,16	0,045
Sex	Male	45 (47.8%)	40 (42.5%)	0,559
	Female	49 (52,2%)	54 (57.5%)	

IOP= intraocular pressure; SD= standard deviation.

On the basis of the genotypes CC and CT/TT of *TXNRD2* rs35934224, the glaucoma group was categorized according to baseline characteristics. The analysis between the groups did not show statistically significant differences (Table 2).

**Table 2:** Baseline characteristics of the patients in the glaucoma group stratified according to *TXNRD2* gene (rs35934224) genotype

Characteristic	Genotypes of the TXNRD2 gene (rs35934224)		
	CC n=68, n (%)	CT/TT n=26, n (%)	p value
Demographic			
Age (years), mean ± SD	68.8 (± 10.4)	70.45 (± 10.8)	0.532
Male	34 (50%)	11 (42.33%)	0.446
Female	34 (50%)	15 (57.76%)	
Medical history			
Familial history of glaucoma	30 (44.1%)	9 (34.6%)	0.318
Smoking	5 (7.3%)	4 (15.4%)	0.236
Diabetes mellitus	13 (19.1%)	6 (23.0%)	0.669
Hypertension	34 (50%)	16 (61.5%)	0.316
Glaucoma indexes, mean ± SD			
IOP in mmHg	18.3 (± 6.0)	15.5 (± 3.0)	0.099
Cup-to-disk ratio	0.81 (± 0.2)	0.84 (± 0.1)	0.368
No. of anti-glaucoma medication	2.12 (± 1.2)	1.96 (± 1.0)	0.705
TREC (rs35934224) genotype	29 (42.6%)	6 (23.1%)	0.175

SD= standard deviation; IOP= intraocular pressure; TREC= trabeculectomy.

Table 3 summarizes the allelic and genotype frequencies of *TXNRD2* rs35934224 in the individuals with POAG and controls. The patients in the glaucoma group presented a higher frequency of the *CC* genotype than the control group (72.3% vs. 59.5%). In the comparison of the *CT/TT* and *CC* genotypes, the combination *CT/TT* presented a higher frequency in the control group than in the case group; however, these results were not statistically significant (40.5% vs. 27.7%; p=0.032; OR=0.56, 95% CI = 0.30-1.03). When evaluating only the *CT* × *CC* genotype, *CT* was associated with protection against POAG (38.2% vs. 24.4%, p=0.022, OR=0.52, 95% CI=0.27-0.98). Allelic distribution was not significantly associated.

**Table 3:** Allele and genotype frequencies of the TXNRD2 in the patients with POAG and controls

	POAG group	Control group (n = 94)	Odds ratio, 95% Cl	p value^*^
TXNRD2 (rs35934224)				
Genotypes				
CC	68 (72.3%)	56 (59.5%)	Reference	Reference
CT	23 (24.4%)	36 (38.2%)	0.52 (0.27-0.98)	0.02
TT	3 (3.3%)	2 (2.3%)	1.23 (0.19-7.65)	0.59
CT/TT	26 (27.7%)	38 (40.5%)	0.56 (0.30-1.03)	0.03
Alleles				
C	159 (84.6%)	148 (78.7%)	0.67 (0.39-1.14)	0.07
T	29(15.4%)	40 (21.3%)		

## DISCUSSION

In the present study, we found a significant association between the CT genotype in the *TXNRD2* gene (rs35934224) with protection to POAG in a Brazilian admixed population^(15)^. On the other hand, we observed a higher frequency of the CC genotype in the POAG group than in the control group.

Oxidative stress and antioxidant systems are important in ocular tissue. Several studies have demonstrated a significant contribution of oxidative stress to the POAG pathogenesis^(16-21)^. In addition, a recent meta-analysis demonstrated an overall increase in oxidative stress molecules in glaucoma, both in the serum and aqueous humor. On the other hand, the authors observed decreases in the levels of some antioxidant markers, which suggests that reduction in ROS could be neuroprotective^(18)^. The mitochondrial thioredoxin reductase (TXNRD) had a central importance for mitochondrial scavenging of ROS^(22)^. It is well established that antioxidant molecules could be implicated in the pathogenesis of glaucoma because oxidative stress might play a causal role in the disease^(18,23,24)^. In addition, polymorphisms in molecules associated to antioxidant systems^(25)^ have been reported in patients with POAG^(11,26,27)^.

Herein, the CT genotype was associated with protection from POAG, while the CC genotype was more prevalent in the POAG group. Our results corroborate, at least in part, the association found in the GWAS study performed by Bailey et al.^(6)^. In this study, they found a significant association between the rs35934224 allele T with protection from POAG (OR 0.77), besides demonstrating the *TXNRD2* expression in retinal ganglion cells and the optic nerve head in normal human ocular tissue^(6)^.

In addition, other SNPs in *TXNRD2* have been associated with POAG in different populations. Bonnemaijer et al. showed the association of the rs16984299 in *TXNRD2* with POAG in African populations^(28)^. Moreover, another study observed a significant association between rs17534001 in the *TXNRD2* gene and POAG in European populations^(29)^.

The role of TXNRD2 in the glaucoma pathogenesis has been evidenced through an experimental glaucoma model in which overexpression of thioredoxin 2, the substrate of thioredoxin reductase 2, increased retinal ganglion cell survival^(29)^. Thus, reduction of ROS levels by TXNRD2 could prevent glaucoma. In addition, the SNP rs35934224 is located in an inter-exon region and is in linkage disequilibrium with other SNPs in regulatory regions such as the 3’-UTR and promoter regions; thus, this nucleotide change may affect the expression levels of the molecule.

In summary, our data suggest the association between the *TXNRD2* gene polymorphism (rs35934224) and POAG in an admixed Brazilian population. However, our study has some limitations such as the small number of subjects enrolled and the lack of functional characterization of TXNRD2. However, we believe that the preliminary results reported herein open new perspectives for the evaluation of the gene in Latin American populations. Further cross-sectional studies with larger cohorts in different populations are necessary to elucidate the potential use of TXNRD2 in clinical practice.
